# Primary Care Physicians’ Knowledge and Self-Perceived Competence Regarding Chronic Kidney Disease Management in Jazan Province, Saudi Arabia: A Questionnaire-Based Cross-Sectional Study

**DOI:** 10.3390/healthcare14142140

**Published:** 2026-07-16

**Authors:** Mostafa Mohrag, Ali Someili, Omar Oraibi, Abdulrahman Mohammed Hakami, Luai Alhazmi, Mohammed Somaili, Mohammed Ali Madkhali, Abdulrahman Khormi, Erwa Elmakki, Mohammed Abdulrasak

**Affiliations:** 1 Department of Internal Medicine, College of Medicine, Jazan University, Jazan 45142, Saudi Arabia; mmohrag@jazanu.edu.sa (M.M.); asomeili@jazanu.edu.sa (A.S.); ooraibi@jazanu.edu.sa (O.O.); ayhakame@jazanu.edu.sa (A.M.H.); lmalhazmi@jazanu.edu.sa (L.A.); misomaili@jazanu.edu.sa (M.S.); mmedkhali@jazanu.edu.sa (M.A.M.); eelmakki@jazanu.edu.sa (E.E.); 2 College of Medicine, Prince Sattam University, Al Kharj 16273, Saudi Arabia; aa.khormi@psau.edu.sa; 3 Department of Clinical Sciences, Lund University, 203 13 Malmo, Sweden; 4 Department of Gastroenterology and Nutrition, Skåne University Hospital, 205 02 Malmo, Sweden

**Keywords:** chronic kidney disease, primary health care, physician knowledge, Saudi Arabia, clinical competence

## Abstract

**Background and aim**: Primary care physicians are central to the early detection and management of chronic kidney disease (CKD), yet knowledge gaps in this setting remain a global concern. This study assessed CKD-related knowledge and self-reported competence among primary healthcare physicians in Jazan Province, Saudi Arabia. **Methods**: A cross-sectional survey was administered to 233 physicians. Knowledge was evaluated using an 11-item questionnaire, and self-reported confidence was measured using a numerical rating scale. **Results**: The median number of correctly answered knowledge questions was 5 out of 11 (IQR 4–6), reflecting considerable variability in knowledge levels across the study population. While most participants correctly identified diabetes as the leading cause of CKD (91%) and recognized its asymptomatic nature (82.8%), significant deficiencies were identified in nephrology referral criteria (35.6%), microalbuminuria-based diagnosis (50.2%), and metformin discontinuation thresholds (58.8%). Notably, only 6.4% correctly identified finerenone as an approved agent for reducing CKD progression in diabetic kidney disease. The median self-reported confidence score was 7 (IQR 5–8). Higher confidence was significantly associated with greater CKD patient exposure (*p* = 0.001) and with higher knowledge scores (τ = 0.207; *p* < 0.0001). **Conclusions**: These findings highlight critical gaps and variability in CKD knowledge among primary care physicians and underscore the need for targeted educational interventions and system-level support to improve early CKD detection and management.

## 1. Introduction

Approximately 850 million individuals worldwide are affected by chronic kidney disease (CKD), with around 4 million currently undergoing kidney replacement therapy [1]. CKD has a global prevalence estimated at 11–13% [2]. Beyond its high prevalence and economic burden, CKD is a leading cause of morbidity and mortality worldwide. Between 1990 and 2017, mortality attributable to CKD increased by 41.5%, elevating it from the 17th to the 12th leading cause of death globally. CKD directly or indirectly accounted for approximately 4.6% of all global deaths, a proportion that continues to rise [3]. CKD affects individuals across all age groups and is associated with increased morbidity, mortality, healthcare expenditure, and impaired quality of life [4].

In recent decades, CKD has emerged as a major public health challenge in Saudi Arabia, characterized by a rising incidence and prevalence of end-stage renal disease (ESRD) [5]. The age-standardized prevalence of CKD stages 1–5 (excluding dialysis patients) in Saudi Arabia has been estimated at 9892 per 100,000 individuals, exceeding rates reported in Western Europe and North America [6]. As of 2021, more than 20,000 patients were receiving dialysis, and 9810 were being followed after kidney transplantation. The overall prevalence of renal replacement therapy (RRT) in Saudi Arabia is estimated at 294.3 per million population [7]. To date, only two studies have examined CKD prevalence within Saudi Arabia. One study involving a relatively young population (mean age 37.4 ± 11.3 years) reported a CKD prevalence of approximately 5.7% [6]. Another study by Mousa et al. found a CKD prevalence of 13.8% among first-degree relatives of hemodialysis patients, with rates two to four times higher in the southern region of the country, where Jazan Province is located [7].

CKD typically progresses silently, with many patients remaining asymptomatic until advanced stages, often after substantial loss of kidney function. It is estimated that approximately 90% of individuals with CKD are unaware of their diagnosis [3]. Consequently, early detection and prevention through screening programs in primary healthcare settings are critical. Early identification has been shown to slow disease progression, reduce cardiovascular morbidity and mortality, and lower overall healthcare costs [8,9]. CKD frequently coexists with other chronic conditions such as diabetes mellitus, hypertension, and obesity, further underscoring the pivotal role of primary healthcare physicians in its early detection and management [10].

Despite this, data assessing CKD knowledge among primary healthcare physicians in Saudi Arabia remain scarce. International studies addressing this issue are limited, but consistently demonstrate significant gaps in CKD-related knowledge among primary care physicians [11,12,13,14,15]. Given that early diagnosis and management of CKD depend heavily on primary care physicians’ understanding of the disease, this study aimed to evaluate the level of CKD knowledge and self-reported competence among primary healthcare physicians in Jazan Province, Saudi Arabia, and to identify areas where further education may be required.

## 2. Materials and Methods

### 2.1. Study Design, Setting, and Participants

This cross-sectional study was conducted and reported in accordance with the Strengthening the Reporting of Observational Studies in Epidemiology (STROBE) guidelines. The study was conducted between October and December 2023 in Jazan Province, located in the southwest of Saudi Arabia, with a population of approximately 1.7 million.

Eligible participants were licensed primary healthcare physicians, including general practitioners and family medicine physicians, who were actively providing outpatient care in Ministry of Health primary healthcare centers in Jazan Province during the study period. Physicians working exclusively in administrative positions, those not involved in direct patient care, those with nephrology subspecialty training, and those who declined participation were excluded.

### 2.2. Sample Size

The target population comprised approximately 500 primary healthcare physicians working under the Ministry of Health in the Jazan Health Directorate. A formal sample size calculation was performed because of the descriptive nature of the primary objective of the study, i.e to estimate CKD-related knowledge and self-perceived competence among primary healthcare physicians in this finite regional workforce. Sample size estimation was based on the formula for cross-sectional studies with finite populations:

Sample size = [1 + {([Z^2^p(1 − p)]/d^2^ − 1)/Population}]

Assuming an expected proportion of adequate CKD knowledge of 50%, a conservative mathematical assumption used to maximize the required sample size in the absence of prior local estimates, with a 95% confidence level (Z = 1.96) and a 5% margin of error, the calculated minimum sample size was 218 physicians. The 50% value was used solely for sample size estimation and was not intended to classify participants as having adequate or inadequate knowledge. To account for an anticipated non-response rate of approximately 15%, a total of 255 physicians were targeted for recruitment.

### 2.3. Sampling Strategy, Questionnaire and Scoring System

Participant recruitment was conducted through a sector-based administrative approach within the Ministry of Health primary healthcare network in Jazan Province. The Jazan Health Directorate officially communicated with the directors of all healthcare sectors in the province, who subsequently forwarded the study invitation to Ministry of Health primary healthcare centers within their respective sectors. Directors of the primary healthcare centers were requested to invite all physicians who met the study eligibility criteria to participate. Thus, recruitment aimed to reach eligible primary healthcare physicians across the province through the existing Ministry of Health administrative structure, rather than through a public database or centralized list of individual physicians. Participation was voluntary, and responses were collected anonymously using an English-language, online self-administered questionnaire distributed via Google Forms™ (Supplementary Materials).

The questionnaire was adapted from a previously published and validated survey [15] and consisted of two sections. The first section collected demographic and professional characteristics, including years of experience, current position, and the average number of CKD patients seen per week. The second section assessed CKD knowledge and competence and included a self-reported confidence score on a scale from 0 to 10.

The knowledge assessment consisted of 11 questions, including both single- and multiple-answer formats, covering CKD diagnostic criteria, albuminuria, risk factors, clinical features, referral thresholds, eGFR interpretation, and management of early-stage CKD. Given the high prevalence of diabetes in the region, one item assessed knowledge regarding metformin discontinuation based on eGFR and another assessed awareness of newer kidney-protective therapies. Each question was awarded one point only if answered completely correctly, yielding a maximum possible score of 11. As this was a physician-level questionnaire-based study rather than a patient-level epidemiological study, patient-level CKD clinical data were not collected. CKD-related exposure was assessed by asking physicians to estimate the number of CKD patients they encountered per week.

### 2.4. Pilot Study

Prior to formal distribution, a pilot study was conducted with 15 primary healthcare physicians who were not included in the final analysis. The pilot aimed to ensure clarity, comprehensibility, and appropriate completion time of the questionnaire.

### 2.5. Data Analysis

Data were analyzed using SPSS software 25. As normal distribution could not be assumed, results are presented as medians with interquartile ranges (IQRs). Group comparisons were performed using the Mann–Whitney U test for two groups and the Kruskal–Wallis test for more than two groups. Associations between continuous variables were assessed using Kendall’s tau correlation coefficient. Statistical significance was set at *p* < 0.05. Question-by-Question performance was extracted from the Google Forms ^TM^ questionnaire.

### 2.6. Ethical Considerations

Ethical approval was obtained from the Jazan Health Ethics Committee, Ministry of Health, Saudi Arabia (approval number: 2385). Informed consent was obtained from all participants. Confidentiality and anonymity were strictly maintained, and no identifiable personal data were collected.

## 3. Results

### 3.1. Participant Characteristics

A total of 233 physicians participated, of whom 141 (60.5%) were male. The majority were general practitioners (*n* = 136; 58.4%), with fewer than five years of professional experience (*n* = 79; 33.9%). Most participants (*n* = 146; 62.7%) reported seeing fewer than 10 CKD patients per week. Non-Saudi physicians constituted 61.4% (143) of the sample. Detailed participant characteristics are presented in Table 1.

The median self-reported confidence score was 7 (IQR 5–8). Confidence scores did not differ significantly by gender (*p* = 0.654), nationality (*p* = 0.668), or years of experience (*p* = 0.956). However, higher exposure to CKD patients was associated with significantly higher confidence scores (*p* = 0.001).

### 3.2. CKD Knowledge Assessment

**Figure 1:**
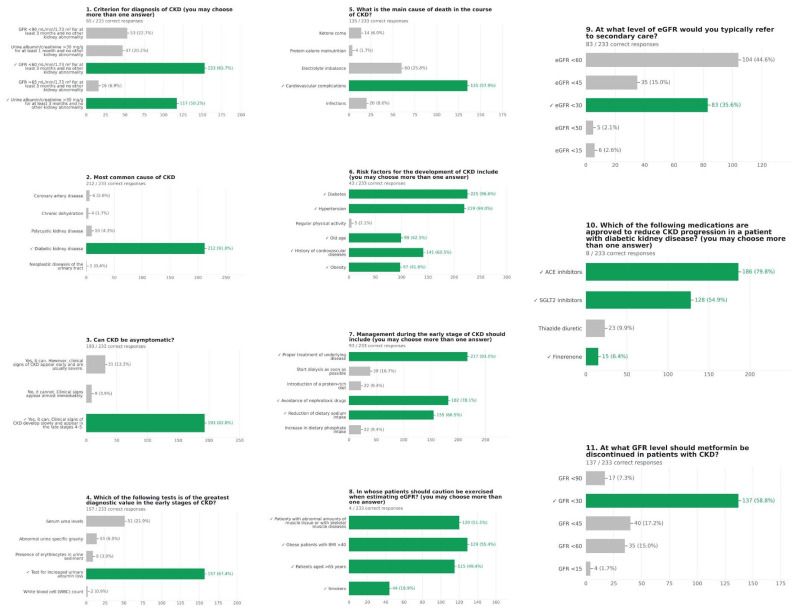
Detailed Question-by-Question Performance in the Participating Physician Cohort.

Overall CKD knowledge was assessed using an 11-question test (Supplementary Materials). The median number of correctly answered questions was 5 (IQR 4–6). Only one participant (0.4%) achieved a score of 10. While 65.7% of participants correctly identified the eGFR-based diagnostic criteria for CKD, only 50.2% correctly identified microalbuminuria criteria. Most participants correctly recognized diabetes as the leading cause of CKD (91%) and acknowledged the predominantly asymptomatic nature of the disease (82.8%). However, only 35.6% correctly identified nephrology referral thresholds based on eGFR, and 58.8% identified the appropriate eGFR cutoff for metformin discontinuation. Detailed question-by-question performance is presented in Figure 1.

Total knowledge scores did not differ significantly by gender (*p* = 0.412) or years of experience (*p* = 0.552). A trend toward higher scores was observed among physicians with greater CKD exposure (*p* = 0.060) and among Saudi physicians (*p* = 0.057), although these did not reach statistical significance. Higher confidence scores were positively correlated with higher knowledge scores (τ = 0.207; *p* < 0.0001).

## 4. Discussion

In this cross-sectional study assessing knowledge and self-reported competence regarding CKD among primary healthcare physicians in Jazan Province, we identified substantial variability and notable gaps in CKD-related knowledge, despite relatively high subjective confidence levels. The median number of correctly answered knowledge questions was 5 out of 11 (IQR 4–6), reflecting considerable heterogeneity in knowledge levels across the study population, consistent with the variability in CKD knowledge reported among primary care physicians in prior studies [16,17]. While most participants correctly recognized diabetes as the leading cause of CKD (91%) and acknowledged its often asymptomatic course (82.8%), consistent with previously reported findings [17], deficiencies were particularly evident in nephrology referral criteria, with only 35.6% correctly identifying eGFR-based referral thresholds. With regards to albuminuria-based diagnosis, only 50.2% correctly identified microalbuminuria criteria, while for medication safety only 58.8% identified the appropriate eGFR cutoff for metformin discontinuation. Performance on the question assessing caution when estimating GFR was strikingly poor, with only 4 out of 233 participants (1.7%) providing a fully correct response, highlighting a critical gap in the nuanced application of renal function assessment in clinical practice. Furthermore, recognition of approved pharmacological agents for reducing CKD progression in diabetic kidney disease was variable; while ACE inhibitors were correctly identified by 79.8% of participants and SGLT2 inhibitors by 54.9%, only 6.4% identified finerenone as an approved agent, reflecting a potential lag between emerging guideline recommendations and clinical education at the primary care level. This is consistent with findings from Madinah, Saudi Arabia, where, although approximately two-thirds of physicians were aware of the five stages of CKD, only 16% correctly identified that patients with Stage 4 CKD should be referred to a nephrologist [18]. Notably, only 15.4% of our respondents were fully confident in CKD screening and diagnosis, a finding that stands in sharp contrast to a Polish study of primary care physicians in which 78.4% correctly identified the diagnostic criterion for CKD [15]. The near-universal recognition of hypertension and diabetes mellitus as CKD risk factors observed in prior studies [17] was similarly reflected in our cohort. These domains are critical for early CKD detection and prevention of disease progression, as kidney disease can be prevented and progression to end-stage renal disease slowed through early detection, lifestyle modification, and appropriate treatment [19,20].

Our findings are consistent with prior international studies demonstrating suboptimal CKD knowledge among primary care physicians, including reports from Europe, South Asia, and sub-Saharan Africa [11,12,13,14,15]. In contrast, a related European cross-sectional survey evaluating self-rated knowledge and competence in CKD management among primary care professionals found that only about one-third of respondents reported being fully confident in CKD management [21], a proportion notably lower than the confidence levels observed in our cohort. Importantly, CKD represents a major cause of morbidity and mortality and constitutes a significant risk factor in the context of hypertension and diabetes [21]. Furthermore, although evidence from Poland indicates that family physicians have a reasonable understanding of the causes, risk factors, and progression of CKD, the same study concluded that additional education and more factual knowledge remain necessary within this profession [15]. Similar deficits in referral thresholds and interpretation of eGFR have been repeatedly described in the literature, with ongoing debate surrounding the optimal criteria—whether risk-based or GFR threshold-based—for nephrology referral in CKD [22]. This convergence suggests that the observed knowledge gaps are not unique to Saudi Arabia but rather reflect a broader, global challenge in equipping primary care systems to manage increasingly complex chronic diseases such as CKD.

The relatively modest overall knowledge scores observed in this study should be interpreted within the context of the structure and demands of the primary healthcare system. Primary healthcare physicians play a critical role in the management of CKD [23]. International evidence points towards the benefit of stronger investment at the primary care level to achieve better chronic disease outcomes and lower long-term healthcare costs; primary care-based health systems have been associated with lower hospitalization rates, less duplication of treatment, more appropriate use of technology, lower healthcare spending, higher quality of care, and reduced healthcare disparities [24], whereas under-resourced primary care settings are associated with delayed diagnosis and increased reliance on tertiary care.

An interesting observation in our cohort was a trend toward higher CKD knowledge scores among Saudi physicians compared with their non-Saudi counterparts, although this difference did not reach statistical significance (*p* = 0.057) and should therefore be interpreted cautiously. This non-significant trend may reflect differences in training standardization and access to locally aligned educational pathways, rather than differences in individual capability; however, this cannot be confirmed from the present data. Saudi physicians may be more likely to undergo nationally regulated training programs with structured exposure to local clinical guidelines and continuing medical education opportunities. In contrast, non-Saudi physicians may enter practice through more heterogeneous international training pathways with variable emphasis on CKD management. Importantly, this observation supports the need for uniform, system-wide educational interventions that ensure consistent baseline competence across the entire primary care workforce.

Overall, CKD knowledge did not significantly vary by gender (*p* = 0.412) or years of experience (*p* = 0.552), and confidence likewise showed no significant differences by gender (*p* = 0.654), nationality (*p* = 0.668), or years of experience (*p* = 0.956). This suggests that knowledge and confidence gaps are broadly distributed across the primary care workforce, independent of demographic factors. Notably, higher subjective confidence scores were positively associated with higher knowledge scores (τ = 0.207; *p* < 0.0001), although confidence alone is insufficient to guarantee adequate competence. Similar findings have been reported regionally; a recent study from the Eastern Province of Saudi Arabia found that most primary care providers expressed high confidence in their ability to use and interpret the urine albumin–creatinine ratio and in their knowledge of appropriate treatment steps, mirroring the pattern of elevated self-assessed competence seen in our cohort [25]. In contrast, another recent Saudi study found that confidence in CKD management varied considerably among healthcare professionals and was shaped by factors such as professional role and years of experience, with diabetologists and those with 3–10 years of experience reporting notably higher confidence levels [26]. In our cohort, however, confidence scores did not differ significantly by years of experience (*p* = 0.956), suggesting that factors beyond experience alone—such as case exposure and structured training—may be more determinative of confidence levels. Indeed, higher exposure to CKD patients was associated with significantly higher confidence scores in our study (*p* = 0.001), suggesting that experiential learning plays an important role. This discordance between perceived and actual knowledge is well recognized in medical education, reflecting a phenomenon whereby trainees and clinicians may overestimate their own competence in areas where their knowledge is limited [27], and reinforces the importance of objective educational reinforcement rather than reliance on self-assessment alone.

**Figure 2:**
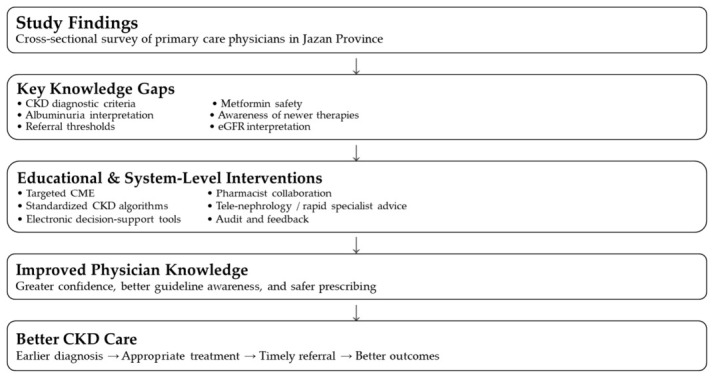
Practical Strategies to Improve Chronic Kidney Disease Management in Primary Care.

Several practical and scalable strategies (Figure 2) could address the deficiencies identified in this study. First, targeted continuing medical education (CME) activities focused on CKD detection, referral criteria, medication safety, and awareness of emerging pharmacological agents such as finerenone should be implemented for primary care physicians regardless of seniority or training background. Evidence supports the effectiveness of such interventions; a review of CME activities found that despite heterogeneity across studies, the majority (79%) demonstrated that CME was associated with meaningful improvements in physician knowledge [28]. Second, the integration of automated eGFR reporting with embedded prescribing alerts for nephrotoxic medications represents a low-cost, system-level safeguard that reduces reliance on individual recall and promotes safer prescribing; the advent of automated eGFR reporting provides clinicians with a simple, readily available, and more accurate measure of renal function to guide prescribing decisions in patients with CKD [29]. Third, establishing rapid-access or virtual nephrology consultation pathways, including telephone or electronic advice services, may empower primary care physicians to make timely referral decisions and manage early-stage CKD more effectively while reducing unnecessary specialist visits. Evidence suggests that telemedicine applied in nephrology has demonstrated non-inferiority to traditional care and has been well accepted by both healthcare professionals and patients [30].

Fourth, incentivizing high-quality CKD care through recognition of guideline adherence, quality improvement initiatives, or CME-linked performance metrics may further encourage engagement with CKD management principles. Evidence underscores the pivotal role of sustained incentives in maintaining quality of chronic disease care; a controlled interrupted time-series study of primary care in Scotland found that withdrawal of a major financial incentive scheme led to significant declines within three years in the recording and management of chronic conditions, including blood pressure control and diabetic foot screening [31]. Fifth, clinical pharmacist involvement may be a practical adjunct, as pharmacist-led CKD interventions have been associated with improvements in selected clinical and patient-centered CKD outcomes [32,33]. Such approaches should focus on supporting clinical decision-making rather than penalizing knowledge deficits, reinforcing a collaborative model of care between primary and specialist services. Evidence supports this approach, as most primary care physicians and nephrologists have been shown to favor collaborative care models for patients with progressive CKD, with such models explicitly incorporating primary care physicians, who have been shown to improve patients’ clinical outcomes [34].

This study has several limitations. Its cross-sectional design precludes causal inference, and knowledge assessment was based on a questionnaire rather than observed clinical behavior. Additionally, the study was conducted in a single region, which may limit generalizability to other parts of Saudi Arabia and the world. Furthermore, because this study represented a physician-level knowledge survey rather than a patient-level epidemiological study, we did not collect clinical or exposure-related data from patients with CKD. Therefore, the potential influence of age, comorbidities, environmental exposures, or medication-related factors on CKD burden in Jazan Province could not be assessed in this study. Nevertheless, strengths include a robust sample size, use of a previously published assessment tool, and focus on clinically meaningful knowledge domains with direct implications for patient outcomes.

## 5. Conclusions

In conclusion, our findings demonstrate important gaps in CKD knowledge among primary healthcare physicians, particularly in areas most relevant to early detection and safe management. Addressing these gaps will require not only educational initiatives but also system-level investment in primary care infrastructure, decision-support tools, and integrated care pathways. Strengthening CKD management at the primary care level represents a critical opportunity to reduce disease progression, improve patient outcomes, and mitigate the growing burden of CKD on healthcare systems.

## Figures and Tables

**Table 1:** Characteristics of Participating Physicians.

**Variable.**		***n*** (**% or IQR**)
**Gender**	Male	141 (60.5)
	Female	92 (39.5)
	Age [Median (IQR)]	37 (33–45)
**Career stage**	Family medicine consultant	17 (7.3)
	Family medicine resident	37 (15.9)
	Family medicine senior specialist	18 (7.7)
	Family medicine specialist	25 (10.7)
	General practitioner	136 (58.4)
**Years of experience**	0–5 years	79 (33.9)
	6–10 years	72 (30.9)
	11–15 years	52 (22.3)
	>15 years	30 (12.9)
**CKD patient visits per week**	0	67 (28.8)
	<10	146 (62.7)
	10–20	5 (2.1)
	>20	15 (6.4)
**Nationality**	Saudi	90 (38.6)
	Non-Saudi	143 (61.4)

## Data Availability

The data presented in this study are available on request from the corresponding author.

## Supplementary Materials

The following supporting information can be downloaded at: https://www.mdpi.com/article/10.3390/healthcare14142140/s1.
