# Supplementary material for: Primary Care Physicians’ Knowledge and Self-Perceived Competence Regarding Chronic Kidney Disease Management in Jazan Province, Saudi Arabia: A Questionnaire-Based Cross-Sectional Study
Source: Healthcare (Basel). 2026 Jul 16;14(14):2140. doi: 10.3390/healthcare14142140 (PMC13409741; doi:10.3390/healthcare14142140)
Supplement: Supplementary file 1 [file healthcare-14-02140-s001.zip › healthcare-4382016-supplementary.pdf]

Primary Care Physicians’ Knowledge and Competence of Chronic Kidney Disease Management in Jazan Region

The fellowing is an anonymous research survey about knowledge and competence of chronic kidney disease (CKD) management among primary care doctors in Jazan region.

The survey takes 5-7 minutes to be completed. This survey dose not acquire private informations from the participants and your confidentiality will be maintained.

If you voluntarily choose to participate in this survey, please confirm your consent below prior to begin the survey.

If you have any questions, please contact:
mmohrag@jazanu.edu.sa

[redacted] Draft saved

* Indicates required question

Email *

☐ Recor[redacted] as the email to be included with my response

I agree to participate in this study and answer the questions as accurate as possible *

◉ Yes

○ No

Primary Care Physicians' Knowledge and Competence of Chronic Kidney Disease Management in Jazan Region

[redacted] Switch account

Your email will be recorded when you submit this form

* Indicates required question

Sociodemographic characters:

Gender *

- ◯ Male
- ◯ Female

Nationality *

- ◯ Saudi
- ◯ Non-Saudi

Age in years *

Your answer

Place of work in Jazan region *

- ◯ Southern Health Sector
- ◯ Middle Health Sector
- ◯ Western Health Sector
- ◯ Northern Health Sector
- ◯ Central Health Sector
- ◯ Jabali Health Sector
- ◯ Bani Malik Health Sector
- ◯ Farasan General Hospital and Health Services

Years of experience in the Primary Health Care centers *

- ◯ 0-5 years
- ◯ 6-10 years
- ◯ 11-15 years
- ◯ More than 15 years

Job title *

- ◯ General Practitioner
- ◯ Family medicine resident
- ◯ Family medicine specialist
- ◯ Family medicine senior specialist
- ◯ Family medicine consultant

The estimated number of patients with CKD who visit your clinic per week for any reason *

- ◯ None
- ◯ Less than 10
- ◯ 10-20
- ◯ More than 20

Back Next Clear form

How confident are you: *

	Not confident about this subject	Some degree of confidence, would like to know more	Confident to practice in this area with support	Confidence to practice in this area without support	Fully confident in this area and could teach others
at screening for CKD in the population with risk factors	☐	☐	☐	☐	☐
at interpreting eGFR results?	☐	☐	☐	☐	☐
at interpreting urine albumin-creatinine ratio (uACR) results?	☐	☐	☐	☐	☐
in your knowledge of stages of kidney disease according to estimated glomerular filtration rate?	☐	☐	☐	☐	☐
in your knowledge of the criteria for diagnosis of chronic kidney disease (CKD)?	☐	☐	☐	☐	☐
at understanding of how to predict CKD prognosis using albuminuria and estimated GFR categories?	☐	☐	☐	☐	☐

at recognizing the possible signs and symptoms of more advanced CKD?	☐	☐	☐	☐	☐
at identifying and treating the complications of CKD?	☐	☐	☐	☐	☐
at initiating medications that have particular benefit in CKD such as ACE-Is and SGLT2-Is?	☐	☐	☐	☐	☐
at using referral guidelines to refer appropriate patients with CKD to secondary care?	☐	☐	☐	☐	☐
in the overall management of patients with CKD?	☐	☐	☐	☐	☐

On a scale of 1 to 10, with 1 being very poor and 10 being excellent, how would you rate your knowledge about chronic kidney disease (CKD)? *

- ◯ 1 very poor
- ◯ 2
- ◯ 3
- ◯ 4
- ◯ 5
- ◯ 6
- ◯ 7
- ◯ 8
- ◯ 9
- ◯ 10 Excellent

1- Criterion for diagnosis of CKD (you may chose more than one answer): * 1 point

- [ ] GFR < 90 ml/min./1.73m2 for at least 3 months and no other kidney abnormality
- [ ] Urine albumin/creatinine >30 mg/g for at least 1 month and no other kidney abnormality
- [ ] GFR < 60 ml/min./1.73m2 for at least 3 months and no other kidney abnormality
- [ ] GFR = 65 ml/min./1.73m2 for at least 3 months and no other kidney abnormality
- [ ] Urine albumin/creatinine >30 mg/g for at least 3 months and no other kidney abnormality

2- Most common cause of CKD * 1 point

- ○ Coronary artery disease
- ○ Chronic dehydration
- ○ Polycystic kidney desease
- ○ Diabetic kidney disease
- ○ Neoplastic diseases of the urinary tract

3- Can CKD be asymptomatic? * 1 point

- ○ Yes, it can. However, clinical signs of CKD appear early and are usually severe.
- ○ No, it cannot. Clinical signs appear almost immediately.
- ○ Yes, it can. Clinical signs of CKD develop slowly and appear in the late stages 4-5.

4- Which of the following tests is of the greatest diagnostic value in the early stages of CKD? * 1 point

- ◯ Serum urea levels
- ◯ Abnormal urine specific gravity
- ◯ Presence of erythrocytes in urine sediment
- ◯ Test for increased urinary albumin loss
- ◯ White blood cell (WBC) count

5- What is the main cause of death in the course of CKD? * 1 point

- ◯ Ketone coma
- ◯ Protein-calorie malnutrition
- ◯ Electrolyte imbalance
- ◯ Cardiovascular complications
- ◯ Infections

6- Risk factors for the development of CKD include (you may chose more than one answer): * 1 point

- ☐ Diabetes
- ☐ Hypertension
- ☐ Regular physical activity
- ☐ Old age
- ☐ History of cardiovascular diseases
- ☐ Obesity

7- Management during the early stage of CKD should include (you may chose more than one answer): * 1 point

- ☐ Proper treatment of underlying disease
- ☐ Start dialysis as soon as possible
- ☐ Introduction of a protein-rich diet
- ☐ Avoidance of nephrotoxic drugs
- ☐ Reduction of dietary sodium intake
- ☐ Increase in dietary phosphate intake

8- In whose patients should caution be exercised when estimating GFR? (you may chose more than one answer): * 1 point

- [ ] In patients with abnormal amounts of muscle tissue or with skeletal muscle diseases
- [ ] In obese patients BMI>40
- [ ] In patients aged > 65 years
- [ ] In smokers

9- At what level of eGFR would you typically refer to secondary care? * 1 point

- ◯ eGFR < 60
- ◯ eGFR < 45
- ◯ eGFR < 30
- ◯ eGFR < 50
- ◯ eGFR < 15

10- Which of the fellowing medications are approved to reduce CKD progression in a patient with diabetic kidney disease? (you may chose more than one answer): * 1 point

- [ ] ACE-inhibitors
- [ ] SGLT2-inhibitors
- [ ] Thiazide diuretic
- [ ] Finerenone

11- At what GFR level should Metformin be discontinued in patients with CKD? * 1 point

- ◯ GFR < 90
- ◯ GFR < 30
- ◯ GFR < 45
- ◯ GFR < 60
- ◯ GFR < 15

Back Submit Clear form
